# Investigation on circular asymmetry of geographical distribution in cancer mortality of Hiroshima atomic bomb survivors based on risk maps: analysis of spatial survival data

**DOI:** 10.1007/s00411-012-0402-4

**Published:** 2012-02-03

**Authors:** Tetsuji Tonda, Kenichi Satoh, Keiko Otani, Yuya Sato, Hirofumi Maruyama, Hideshi Kawakami, Satoshi Tashiro, Masaharu Hoshi, Megu Ohtaki

**Affiliations:** 1 Department of Environmetrics and Biometrics, Research Institute for Radiation Biology and Medicine, Hiroshima University, 1-2-3 Kasumi, Minami-Ku, Hiroshima, 734-8551 Japan; 2 Division of Radiation Information Registry, Research Institute for Radiation Biology and Medicine, Hiroshima University, 1-2-3 Kasumi, Minami-Ku, Hiroshima, 734-8551 Japan; 3 Department of Epidemiology, Research Institute for Radiation Biology and Medicine, Hiroshima University, 1-2-3 Kasumi, Minami-Ku, Hiroshima, 734-8551 Japan; 4 Department of Cellular Biology, Research Institute for Radiation Biology and Medicine, Hiroshima University, 1-2-3 Kasumi, Minami-Ku, Hiroshima, 734-8551 Japan; 5 Department of Radiation Biophysics, Research Institute for Radiation Biology and Medicine, Hiroshima University, 1-2-3 Kasumi, Minami-Ku, Hiroshima, 734-8551 Japan

**Keywords:** Atomic bomb survivors, Direct exposure, Indirect exposure, Spatial survival data, Spatially varying coefficient

## Abstract

While there is a considerable number of studies on the relationship between the risk of disease or death and direct exposure from the atomic bomb in Hiroshima, the risk for indirect exposure caused by residual radioactivity has not yet been fully evaluated. One of the reasons is that risk assessments have utilized estimated radiation doses, but that it is difficult to estimate indirect exposure. To evaluate risks for other causes, including indirect radiation exposure, as well as direct exposure, a statistical method is described here that evaluates risk with respect to individual location at the time of atomic bomb exposure instead of radiation dose. In addition, it is also considered to split the risks into separate risks due to direct exposure and other causes using radiation dose. The proposed method is applied to a cohort study of Hiroshima atomic bomb survivors. The resultant contour map suggests that the region west to the hypocenter has a higher risk compared to other areas. This in turn suggests that there exists an impact on risk that cannot be explained by direct exposure.

## Introduction

The risk of disease or death caused by exposure to atomic bomb radiation has been evaluated using estimated radiation doses based on information concerning age, shielding conditions, and distance from the hypocenter under the assumption that the radiation dose decreases with increasing distance from the hypocenter (see, e.g., Preston et al. 2007; Matsuura et al. 1997). For details of the dosimetry system used, see for example the DS02 system (Cullings et al. 2006; Young and Kerr 2005). The corresponding risk analyses focused solely on the risk from direct exposure to the atomic bomb, while the risk from indirect exposure due to residual radioactivity has been not evaluated in previous analyses. This means that the geographical distribution of risk has been structurally restricted to concentric circles under the assumption that the influence of direct exposure essentially depends on the distance from the hypocenter. For example, Peterson et al. (1983) have fitted Cox’s proportional hazard models to cancer mortality rates, to investigate circular asymmetry around the hypocenter in Hiroshima and Nagasaki. Gilbert and Ohara (1984) have analyzed data on acute symptoms. They divided the survivors in the Life Span Study (LSS) cohort, registered at the Radiation Effect Research Foundation (RERF), into eight groups according to the survivors’ location at the time of atomic bomb exposure relative to the hypocenter and evaluated the relative risk of each octant compared with that for survivors in the octant of east–north-east direction. However, we consider their approach to be not enough to investigate circular asymmetry around the hypocenter, because they evaluated only relative risks for each octant with respect to the location at exposure relative to the hypocenter and did not consider heterogeneity of risk in each octant.

Recently, survivors suspected of having suffered from indirect exposure were reported by Kamada et al. (2006), Kamada and Kawakami (2008), and Tonda et al. (2008) through biological studies and statistical analyses of the incidence of leukemia among the survivors who entered Hiroshima City on August 6, 1945, after the explosion of the atomic bomb. Furthermore, several questionnaire surveys (Uda et al. 1953; Masuda 1989) showed that so-called Black Rain, which might have included radioactivity, fell around the western part of Hiroshima City and the northwest suburbs for several hours just after the explosion. Ohtaki (2011) demonstrated spatial-time distributions of Black Rain using a nonparametric smoothing method applied to data from a questionnaire survey conducted by Hiroshima City in 2008, of about 37,000 inhabitants of Hiroshima and its suburbs that might have experienced Black Rain.

In the present paper, a statistical method is applied to evaluate the risk with respect to individual location at exposure rather than dose and construct a “risk map,” that is, a map based on the risk evaluated by location, to visually grasp the geographical distribution of risk without structural restrictions. The risk map allows discussing possible effects of indirect exposure due to “Black Rain” and other radioactivity on risk of mortality.

## Materials and methods

### Data

The database of atomic bomb survivors (ABS), registered at the Research Institute for Radiation and Medicine (RIRBM) at Hiroshima University, was used in the present study. The ABS differs from the LSS of the RERF, because the ABS cohort includes examined survivors residing in Hiroshima Prefecture, and data on health status for survivors also have been cumulatively compiled in the database. The extent of overlap between survivors in the ABS and the LSS was examined by Hayakawa et al. (1994) and Hoshi et al. (1996). Hayakawa et al. (1994) showed that the dose estimates of the ABS were close to those of the LSS among the overlapped subjects. However, it has not been tested how they agree to DS02.

**Fig. 1:**
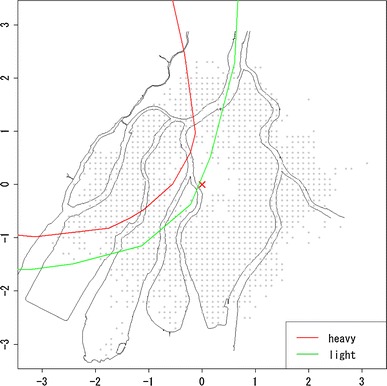
Plot of location at exposure on the map of Hiroshima City, where the *vertical* and *horizontal scales* are the coordinates in units of kilometers with the origin being the hypocenter (*red cross*); *gray* points represent locations of survivors at the time of exposure; *red* and *green lines* represent the boundary of heavy and light rainfall area of “Black Rain” based on Uda’s questionnaire survey

From the ABS, we chose 31,055 subjects for analysis who satisfied the following conditions: (i) being alive and recognized as an atomic bomb survivor as of January 1, 1980 and (ii) having coordinate information on location at the time of atomic bomb exposure (abbreviated in the following as “location at exposure”). These subjects were followed until December 31, 1997. The endpoint is death from solid cancers (number of deaths: 2,545). Subjects were treated alive at the end of follow-up, in case migration and loss to follow-up for other reasons as censoring (number of subjects: 28,510). Mesh coordinates of 100 m in width were used to define location at exposure [Hoshi et al. (1996)]. Sex, age at atomic bomb exposure (abbreviated in the following as “age at exposure”), and shielding condition were used as covariates. Estimations of radiation doses were based on Hoshi et al. (1996) and Matsuura et al. (1997). Figure 1 is the scatter plot of location at exposure with the hypocenter as the origin. Gray lines represent the map of Hiroshima city according to the town planning map made between 1925 and 1928. The vertical and horizontal scales are the coordinates in units of kilometers with the origin being the hypocenter. The red and green lines are boundary of heavy and light rainfall area of “Black Rain” based on Uda’s questionnaire survey (Uda, et al. 1953). Note that upper left region of boundary is rainfall area.

### Statistical analyses

Data containing information on location are called “spatial data.” Several methods for analyzing spatial data have been proposed, depending on the type of outcome. Geographically weighted regression (GWR), proposed by Fotheringham et al. (2002), corresponds to multiple linear regression analysis of spatial data. GWR is essentially repeated local multiple linear regressions applied to data in the neighborhood of a given location. The GWR approach can be extended to logistic regression for spatial binary data and Poisson regression for spatial count data, but the methodology for spatial survival data, such as those in the study of atomic bomb survivors, still remains to be developed. Recently, Tonda and coworkers (Tonda et al. 2010) proposed a statistical method for spatial data by extending a method proposed for longitudinal data (Satoh and Yanagihara 2010; and Satoh et al. 2009). Their approach is applicable not only to spatial continuous and discrete data but also to spatial survival data. In the present paper, a method is developed for estimating the geographical distribution of mortality risk for atomic bomb exposure by extending Cox’s proportional hazards model for spatial survival data (Tonda et al. 2010); the resulting method is applied to a cohort study of Hiroshima atomic bomb survivors.

#### Hazard model with spatially varying coefficients

Consider the proportional hazards model with spatially varying coefficients, which allows the effect of covariates to vary with location. Let (*u*, *v*), *r*, *t*, sex, and atb denote location at exposure, registered age, attained age, gender (sex = 0 if male, sex = 0 if female), and age at exposure. The proportional hazards function with spatially varying coefficient is then given by

1 $$ h\left( {t|u,v,t > r} \right) = h_{0} \left( {t|{\text{shielding}}} \right)\;\exp \left( {\beta_{l} (u,v) + \beta_{s} \times {\text{sex}} + \beta_{a} \times {\text{atb}}} \right) $$

where $h_{0} \left( {t|{\text{shielding}}} \right)$ is the baseline hazard function dependent on the shielding condition, β_*l*_ (*u*, *v*) is the spatially varying coefficient, and β_*s*_ and β_*a*_ are ordinary regression coefficients that are constant with regard to location at exposure. Note that Eq. 1 represents an ordinary Cox model if the spatially varying coefficients are replaced by constant coefficients. Therefore, Eq. 1 represents an extension of a Cox model, and the interpretation of coefficients in Eq. 1 is similar to that with a Cox model. In particular, exp (β_*l*_ (*u*, *v*)) denotes the hazard ratio compared with the location as the reference.

It is assumed that the shape of (β_*l*_ (*u*, *v*)) is in a class described by linear combinations of unknown parameters $\varvec{\theta}$ and known basis functions $\user2{x}\left( {u,v} \right)$: that is,

2 $$ \beta_{l} (u,v) = \varvec{\theta^{\prime}}\user2{x}(u,v) = \sum\limits_{j = 1}^{q} {\theta_{j} x_{j} (u,v)} ,\quad \,\varvec{\theta}= \left( {\theta_{1} , \ldots ,\theta_{q} } \right)^{\prime } ,\quad \, \user2{x}(u,v) = \left( {x_{1} (u,v), \ldots ,x_{q} (u,v)} \right)^{\prime } . $$

We use a polynomial surface basis, which is commonly used in the field of spatial interpolation (Ripley 1981; Venables and Ripley 2002). For example, a quadratic polynomial surface basis is given by

3 $$ \user2{x}(u,v) = \left( {1,u,v,u^{2} ,v^{2} ,uv,u^{2} v,uv^{2} ,u^{2} v^{2} } \right)^{\prime } . $$

In addition, a circular surface basis is expressed by $\user2{x}(u,v) = \left( {1,u^{2} + v^{2} } \right)^{\prime } .$ To obtain a smoother shape for the spatially varying coefficient, one can use, for example, a B-spline or a Gaussian basis. Details are given in Satoh et al. (2003), Ruppert et al. (2003), and Konishi and Kitagawa (2010).

For spatial survival data, $\left\{ {(u_{i} ,v_{i} ),\delta_{i} ,t_{i} ,{\text{sex}}_{i} ,{\text{atb}}_{i} ;\quad \, i = 1, \ldots ,n} \right\}$, where δ_*i*_ denotes the indicator variable specifying whether subject *i* is censored or not at time t_*i*_, with 1 denoting a failure and 0 denoting censored, the unknown parameters $\varvec{\theta}$, β_*s*_ *,* and β_*a*_ can be estimated by maximizing the partial likelihood (Cox 1972, 1975):

4 $$ l(\varvec{\theta },\beta _{s} ,\beta _{a} ) = \prod\limits_{{i \in I}} {\frac{{\exp \varvec{\theta }^{\prime } \user2{x}(u_{i} ,v_{i} ) + \beta _{s}  \times sex_{i}  + \beta _{a}  \times atb_{i} }}{{\sum\nolimits_{{j \in R_{i} }} {\exp \varvec{\theta }^{\prime } \user2{x}(u_{j} ,v_{j} ) + \beta _{s}  \times sex_{j}  + \beta _{a}  \times atb_{j} } }}}, $$

where *I* is the set of indices of failure cases, that is, $I = \{ i;\;\delta_{i} = 1,\;i = 1, \ldots ,n\}$, and *R*_*i*_ is the set of indices of cases who are alive at *t*_*i*_, that is, *R*_*i*_={*j*; *t*_*j*_ > *t*_*i*_ > *r*_*j*_, *j* = *1*, …, *n*}

Let ${\hat{\mathbf{\theta }}}$ denote the estimator of ${\varvec{\theta}}$; the estimator of β_*l*_ (*u*, *v*) is expressed by $\hat{\beta }_{l} (u,v) = {\hat{\mathbf{\theta }}}^{\prime } {\mathbf{x}}(u,v)$. Theoretical properties of $\hat{\beta }_{l} (u,v)$ given in Tonda et al. (2010) allows to construct a confidence region for β_*l*_ (*u*, *v*) and test hypotheses about the shape of β_*l*_ (*u*, *v*). In particular, the test of the hypothesis

5 $$ H_{0} :\beta_{l} (u,v) = {\text{const}} . {\text{ for any }}(u,v) \in R^{2} , $$

is a test of spatial homogeneity. This test is meaningful because it verifies statistically whether β_*l*_ (*u*, *v*) is spatially varying or not. If the hypothesis of spatial homogeneity is rejected, there exists a regional difference on β_*l*_ (*u*, *v*). Any further discussion of the methodology for confidence regions and tests for β_*l*_ (*u*, *v*) is beyond the scope of this paper; for additional information, see Tonda et al. (2010).

#### Dose effect model

If dose denotes radiation dose (Hoshi et al. 1996; Matsuura et al. 1997), the risk for direct exposure from atomic bomb radiation was modeled by adding the term β_*d*_ (t, atb)× dose to the hazard function in Eq. 1, that is,

6 $$ h(t|u,v,t > r) = h_{0} (t)\exp \left( {\beta_{l} (u,v) + \beta_{s} \times {\text{sex}} + \beta_{a} \times {\text{atb}}} \right)\left( {1 + \beta_{d} (t,{\text{atb}}) \times {\text{dose}}} \right), $$

where *h*_0_ (*t*) denotes the common baseline hazard. The coefficient β_*d*_ (*t*, atb) depends on both of attained age and age at exposure. According to a mathematical model of carcinogenesis, such as the generalized Armitage-Doll model (Ohtaki et al. 1985; Pierce and Mendelsohn 1999; Ohtaki and Niwa 2001; Pierce and Vaeth 2003), we assume the functional structure

7 $$ \beta_{d} (t,{\text{atb}}) = {{\left( {\lambda_{d} + \lambda_{a} \times {\text{atb}}} \right)} \mathord{\left/ {\vphantom {{\left( {\lambda_{d} + \lambda_{a} \times {\text{atb}}} \right)} t}} \right. \kern-\nulldelimiterspace} t}, $$

where λ_*d*_ denotes the effect of radiation dose for survivors exposed at age 0 and λ_*d*_ denotes the influence of age at exposure on sensitivity to radiation dose (the derivation of Eq. 7 is given in the “Discussion” section). Equation (7) means that the risk for radiation varies with age at exposure and decreases with increasing attained age. The unknown parameters can be estimated with a slight modification of the partial likelihood in Eq. 4, because $1 + \beta_{d} (t,{\text{atb}}) \times {\text{dose}} \approx \exp \left( {\beta_{d} (t,{\text{atb}}) \times {\text{dose}}} \right)$ is valid for small doses. Note that the hazard function excluding β_*l*_ (*u*, *v*) from Eq. 6 represents the ordinary Cox’s hazard model with time-dependent variables, given by

8 $$ h(t) = h_{0} (t)\exp \left( {\beta_{s} \times {\text{sex}} + \beta_{a} \times {\text{atb}}} \right)\left( {1 + \beta_{d} (t,{\text{atb}}) \times {\text{dose}}} \right). $$

Equation (8) was used for modeling the relationship between the mortality risk and risk from direct exposure in previous studies [see, e.g., Pierce and Vaeth (2003)].

## Results

The proposed method was applied to data from a cohort study of Hiroshima atomic bomb survivors. The method is easy to implement using statistical packages that execute Cox model, such as SAS, SPSS, and R. We used the “survival” package version 2.36-2 in R version 2.12.0 [R Development Core Team (2010)].

Table 1 demonstrates the goodness-of-fit among the various models on a *q*-th order polynomial basis (*q* = 1, 2, 3, 4) and a circular basis to describe the shape of β_*l*_ (*u*, *v*) in Eq. 1. Statistically comparing five models using Akaike’s Information Criteria (AIC; Akaike 1973), we selected the most suitable basis among the five in the manner of variable selection [see Tableman and Kim (2004), chapter 5]. Table 1 suggests the quadratic polynomial model to be optimal. The martingale residuals [Klein and Moeschberger (2003), chapter 11; Therneau and Grambsch (2000), chapter 4] were used to check whether the quadratic polynomial basis adequately describes the geographical distribution of risk. Assessing the presence or absence of spatial trend of residuals by applying generalized additive models [Wood (2006) chapter 4], the hypothesis of spatial homogeneity for residuals in the quadratic polynomial model was not rejected. Therefore, the quadratic basis seems to describe the spatial trend of risk adequately.

**Table 1:** Comparison of goodness-of-fit among five models

Type of basis	Circular	Polynomial			
		*q* = 1	*q* = 2	*q* = 3	*q* = 4
Number of parameters	3	5	10	17	26
AIC	38,009.9	38,039.7	38,004.7	38,010.0	38,016.1

**Fig. 2:**
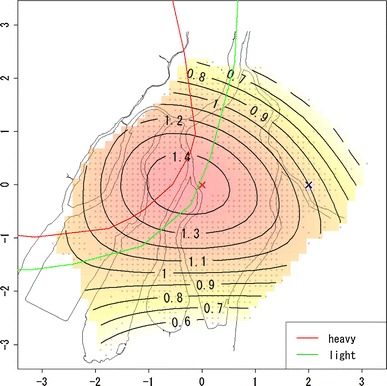
Estimated risk map of mortality based on the quadratic polynomial model. Values on the contours are hazard ratios compared with the reference location (*blue cross*) that is 2 km from the hypocenter to the east. The *red* and *green lines* represent the boundary of heavy and light rainfall area of “Black Rain” based on Uda’s questionnaire survey

Figure 2 shows the estimated risk map of mortality based on the quadratic polynomial model, while Table 2 shows the estimated coefficients of β_*s*_ and β_*a*_. In Fig. 2, the contours on the map represent the hazard ratio, $\exp \left( {\beta_{l} (u,v)} \right)$, for each location compared with the reference location, marked as the blue cross, that is 2 km from the hypocenter toward the east. From Fig. 2, it can be seen that the mortality risk decreases with increasing distance from the hypocenter, but the geographical distribution of the risk map is not concentric: The west area appears to have a higher risk compared with other areas.

**Table 2:** Estimated coefficients for the quadratic polynomial model

Parameter	Estimate	se	*z*	*p*
β_*s*_	0.784	0.041	19.2	<0.001
β_*a*_	−0.087	0.003	−30.4	<0.001

**Fig. 3:**
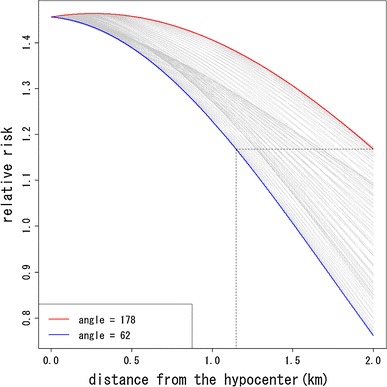
Comparison of decreasing trend of relative risks with distance from the hypocenter by angles of location at exposure. The *red* and *blue curves* denote the highest and lowest trend, whose angles are 178º (about west direction) and 64º (about north–north-east direction)

**Fig. 4:**
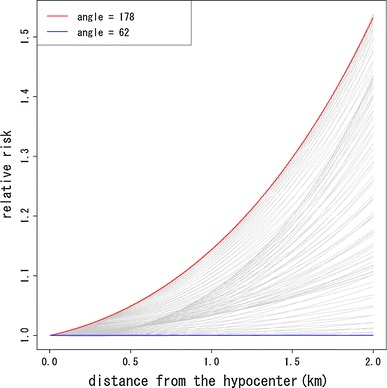
Comparison of increasing trend of relative risks, relative to those at angle 64° (about north–north-east direction), with distance from the hypocenter by angles of location at exposure

**Fig. 5:**
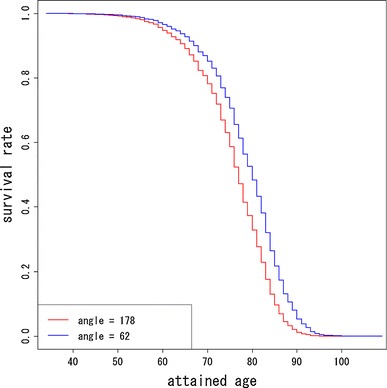
Estimated survival curves for men adjusted by age at exposure and location at exposure. The red and blue curves denote 10 years age at exposure and location at exposure with 2 km from the hypocenter at angles 178° and 62°, respectively

In Fig. 3, the decreasing trend of risk with distance from the hypocenter by direction of location at exposure defined by angle from the hypocenter is compared. Angles 178° (about west direction) and 62° (about north–north-east direction) had highest and lowest relative risks, respectively. Figure 3 also suggests that the risk at 2 km from the hypocenter at angle 178° corresponds to the risk at 1,147 m at angle 62°. Figure 4 shows the differences of relative risks by angle from the hypocenter compared with those of angle 62° (about north–north-east direction). Figure 4 suggests that the differences of relative risks become larger with increasing distance from the hypocenter. Figure 5 shows the estimated survival curves at 2 km from the hypocenter at angles 178° and 62° for women with 10 years age at exposure.

**Fig. 6:**
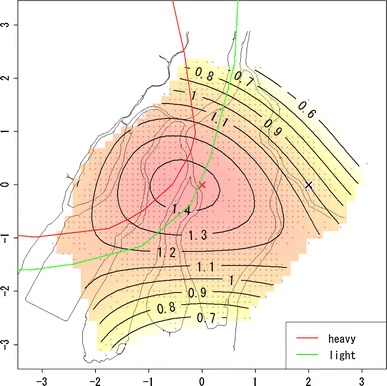
Estimated risk map of mortality based on the quadratic polynomial model with dose adjustment. Values on the contours are hazard ratios compared with the reference location (*blue cross*) that is 2 km from the hypocenter in the east. The *red* and *green lines* represent the boundary of heavy and light rainfall area of “Black Rain” based on Uda’s questionnaire survey

**Fig. 7:**
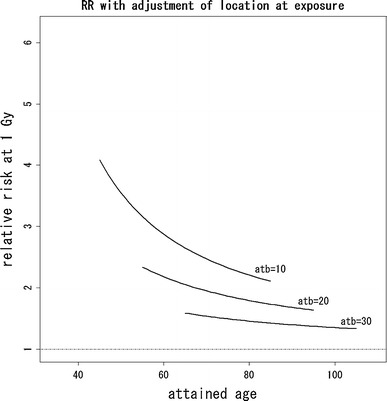
Estimated relative risks with adjustment for location at exposure based on the hazard function in Eq. 6. Each curve denotes the plot of $\exp \left( {\beta_{d} (t,atb)} \right)$, which gives relative risks (RR) at 1 Gy with attained age for ages at exposure 10, 20, and 30 years

**Fig. 8:**
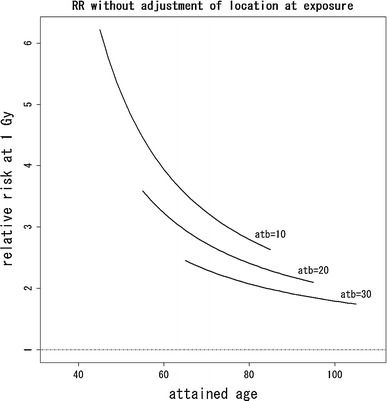
Estimated relative risks without adjustment for location at exposure, based on the hazard function in Eq. 8. Each curve denotes the plot of $\exp \left( {\beta_{d} (t,atb)} \right)$, which gives relative risks (abbreviated “RR”) at 1 Gy with attained age for ages at exposure 10, 20, and 30 years

Finally, we considered removing the risk for the direct exposure from the risk map in Fig. 2 using the dose effect model. The risk for direct exposure can be drawn as a function of radiation dose. In this case, analysis had to be restricted to those individuals for whom information on radiation dose is available, which slightly decreased the number of subjects. The resulting risk map for the dose–effect model with quadratic basis is given in Fig. 6. Table 3 shows the estimated coefficients except for β_*l*_ (*u*, *v*). In Figs. 7 and 8, the estimated relative risks at 1 Gy with attained age for ages at exposure 10, 20, and 30 years based on Eq. 7 are shown. Figure 7 presents estimated relative risks with adjustment for location at exposure based on the hazard function in Eq. 6, with estimated coefficients given in Table 3, while Fig. 8 presents estimated relative risks without adjustment for location at exposure, based on the ordinary Cox model in Eq. 8, with estimated coefficients given in Table 4.

**Table 3:** Estimated coefficients for the dose effect model

Parameter	Estimate	se	*z*	*p*
β_*s*_	0.770	0.046	16.6	<0.001
β_*a*_	−0.087	0.003	−27.0	<0.001
λ_*d*_	79.946	25.727	3.1	0.002
λ_*a*_	−1.662	1.008	−1.6	0.099

**Table 4:** Estimated coefficients for the ordinary Cox model

Parameter	Estimate	se	z	*p*
β_*s*_	0.742	0.046	16.1	<0.001
β_*a*_	−0.088	0.003	−26.2	<0.001
λ_*d*_	94.215	23.437	4.0	<0.001
λ_*a*_	−1.196	0.917	−1.3	0.190

## Discussion

**Fig. 9:**
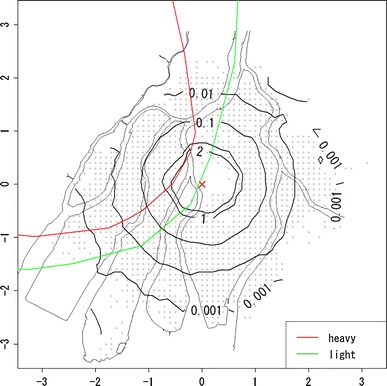
Geographical distribution of location-averaged radiation dose. Values on the contours represent the average of radiation dose (Gy) by location at exposure. The *red* and *green lines* represent the boundary of heavy and light rainfall area of “Black Rain” based on Uda’s questionnaire survey

Figure 9 presents the contour map based on estimated direct radiation dose averaged by location at exposure. From Fig. 9, it can be seen that the geographical distribution of direct radiation dose is close to concentric circles. This means that if the risk due to causes other than the direct exposure was negligible compared with that of direct exposure, then the contours in the risk map should be well approximated by concentric circles. If not, however, the risk contours should be far from concentric and circular. According to Table 1 and Figs. 2, 3, 4, and 5, the resultant risk map for a cohort of Hiroshima atomic bomb survivors (Fig. 2) suggests that the quadratic polynomial contours are suitable indeed, but not concentric circles. This suggests that there existed risk factors other than direct radiation exposure.

We also considered removing risks for direct exposure from the risk map in Fig. 2, in order to grasp the geographical distribution of risks for potential causes other than the direct exposure. This purpose was achieved by adding the term β_*l*_ (*t*, atb)× dose to the hazard function. The age dependence of the dose effect has been formulated under the assumption that tumorigenesis requires multi stages of cell variation and that any carcinogenic transitions of cell variation are sensitive to radiation exposure [for details, see Ohtaki and Niwa (2001)]. According to the generalized Armitage-Doll model (Ohtaki et al. 1985; Pierce and Mendelsohn 1999; Ohtaki and Niwa 2001; Pierce and Vaeth 2003), the hazard function in Eq. 1 is modified by

9 $$ \begin{aligned} h(t|u,v) & = h_{0} (t)\exp \left( {\beta_{l} (u,v) + \beta_{s} \times {\text{sex}} + \beta_{a} \times {\text{atb}}} \right) \\ & \quad \times \left( {1 + {{\alpha_{d} \exp \left( {\alpha_{a} \times {\text{atb}}} \right)} \mathord{\left/ {\vphantom {{\alpha_{d} \exp \left( {\alpha_{a} \times {\text{atb}}} \right)} t}} \right. \kern-\nulldelimiterspace} t} \times {\text{dose}}} \right)^{k - 1} , \\ \end{aligned} $$

where α_*d*_ denotes the risk due to radiation exposure, α_*a*_ the relative sensitivity varying with age at exposure, and *k* the number of mutations required for a normal cell to become malignant. For convenience, the following log-linearization was applied

10 $$ \begin{aligned} \left( {1 + {{\alpha_{d} \exp \left( {\alpha_{a} \times {\text{atb}}} \right)} \mathord{\left/ {\vphantom {{\alpha_{d} \exp \left( {\alpha_{a} \times {\text{atb}}} \right)} t}} \right. \kern-\nulldelimiterspace} t} \times {\text{dose}}} \right)^{k - 1} & \approx 1 + {{(k - 1)\alpha_{d} \left( {1 + \alpha_{a} \times {\text{atb}}} \right)} \mathord{\left/ {\vphantom {{(k - 1)\alpha_{d} \left( {1 + \alpha_{a} \times {\text{atb}}} \right)} t}} \right. \kern-\nulldelimiterspace} t} \times {\text{dose}} \\ & = 1 + {{\left( {\lambda_{d} + \lambda_{a} \times {\text{atb}}} \right)} \mathord{\left/ {\vphantom {{\left( {\lambda_{d} + \lambda_{a} \times {\text{atb}}} \right)} t}} \right. \kern-\nulldelimiterspace} t} \times {\text{dose}}, \\ \end{aligned} $$

where $$ \lambda_{d} \equiv (k - 1)\alpha_{d} $$ and $$ \lambda_{a} \equiv (k - 1)\alpha_{d} \alpha_{a} $$.

Equation (7) is now derived by substituting Eq. 10 into Eq. 9. According to Fig. 6, the resultant risk map, excluding the risks for direct exposure, still has contours skewed toward the west direction. In addition, the test for the hypothesis on spatial homogeneity, formulated by Eq. 5, was rejected (*p* < 0.001). These results may provide further evidence of risks for causes other than direct exposure.

As was mentioned in the introduction, several questionnaire surveys showed that Black Rain, which might have included radioactivity, fell around the western part of Hiroshima city and north–west suburbs for several hours just after the explosion. According to the latest results on the geographical distribution of Black Rain (Ohtaki 2011) and Uda’s rainfall area described in Fig. 2, the area of rainfall appears roughly similar to the region of high risk in Fig. 2. This similarity suggests that Black Rain might be a possible risk factor accounting for the geographical distribution of cancer mortality in Fig. 2. It should be noted, however, that there might be other risk factors affecting mortality such as socioeconomic status, life style, and environmental factors that are probably unrelated to radiation exposure due to the atomic bomb. These factors might correlate through association with particular regions, but this will be difficult check.

Note that Peterson et al. (1983) have also studied the circular asymmetry around the hypocenter in Hiroshima and Nagasaki for the LSS cohort of RERF. They divided the survivors into eight groups by the octants according the survivors’ location at exposure and fitted a Cox’s proportional hazard model. According to their results, the survivors in the west–north–west octant had the highest risk and the relative risk of survivors in the west–north–west compared with those in the east–north–east was about 1.24. As was mentioned in the introduction, their approach suffered from a lack of continuity of risks within groups and between groups. Therefore, they could not grasp any regional spatial trend of risk within and between octants. On the other hand, our results for the ABS cohort of RIRBM can be used to understand the spatial trend visually. Our result in Fig. 3 is roughly consistent with the areas with higher risks in Peterson et al. (1983). In addition, Fig. 4 shows that the differences in the relative risk among angles of location at exposure become larger with increasing distance from the hypocenter, while Peterson et al. (1983) could only evaluate the relative risks by octants. In this sense, our results are somewhat more valuable than those of Peterson et al. (1983).

## Conclusion

The risk map shown in the present work can be interpreted in terms of the radiation dose required to explain the fitted contours for the hazard ratio in Fig. 6. For this, we focused on the hazard ratios at the locations with 2-km distance from the hypocenter in Fig. 6. The relative risk between highest and lowest risk at such locations is about 1.6. This suggests an excess relative risk (ERR) of about 0.6 due to causes other than direct exposure. This value might correspond to quite a large dose (i.e., of more than a Gray) if most of this additional risk is caused by external exposure not yet included in the estimated direct doses, because the ERR per Gray for solid cancer among atomic bomb survivors is on the order of about 0.5 (see, e.g., National Research Council 2006). This is quite unlikely as direct radiation doses where verified experimentally for example by retrospective thermoluminescence measurements on environmental samples (see, e.g., Cullings et al. 2006; Young and Kerr 2005). However, it might be possible that additionally chronic continuous exposure and individual variability caused by internal exposure, that is not included in the current (direct) dose estimates for the atomic bomb survivors, had a large effect on cancer mortality risk among atomic bomb survivors. Unfortunately, data on incorporated radionuclides from fallout are limited, and the effect of any internal exposure requires further clarification. Therefore, the doses corresponding to the contours of risk shown in Fig. 6 also should be an issue in the future. As already mentioned, there might be additional risk factors affecting mortality such as socioeconomic status, life style, and environmental factors that could also explain part of the observed asymmetry, but these factors are difficult to investigate due to limited data available.
